# The Coral Islands and Fiji

**Published:** 1889-02-23

**Authors:** 


### The Coral Islands and Fiji.

The recent request for a lady to offer herself as nurse-matron for a hospital in Fiji has produced such marked evidence of the ignorance that prevails as to this country that we very gladly publish the following account of this delightful group of islands.

The traveller who desires to visit the regions of which I purpose telling the readers of The Hospital would require a somewhat lengthy vacation to fulfil his purpose; still, it is possible, and even easy, for a resident in Great Britain to make a flying trip to the southern seas, and take a glimpse at the wonderful scenes that await and reward those that are sufficiently enterprising to visit them.

Being determined to acquaint myself with the coral islands, I left Auckland, New Zealand, in a snug little schooner of 60 tons, manned by four hands all told, and headed due north, with a gale of wind behind and 1,200 miles of clear sea ahead. No vessels frequent those lonely waters, with the exception of the monthly boat and an occasional schooner carrying a load of merchandise. Never a sail came upon our horizon, but after twelve days' steady progress the morning dawned upon a sight that, once seen, can never be forgotten. On the port bow was the coral island of Tonga-tabu, its white sand lining the water's edge, the bushes and trees growing quite up to the sand, and forming a dark green background. These trees were chiefly cocoa-nuts, tall and graceful, waving their proud heads in the breeze, like monarchs above the rest of creation. As we floated along—for there was now but little wind—we could look down into the water, and see great coral trees growing up from a depth of 30 feet and more, and these seemed to roll beneath us, like a panorama, some pink, some white, some grey, with dolphin and other fish occasionally darting about amongst them.

As the sun rose, life seemed to be awakened on shore, for drums were now being beaten, and the soft full voices of the natives were singing a wild kind of song or chant. When we landed we had many opportunities of examining these denizens of the Sunny South. They are a poetical, dreamy race, fond of infinite leisure and ease, and opposed to labour or exertion of any kind. They rather look down upon the white man, arguing that, with all his culture and refinement, he cannot live without working, and is therefore less able to solve the social problem than themselves.

Strewed about the surface of the water, and clustering around Tonga, are hundreds of little coral islands, varying in size from a square mile to a square yard, and all have their cocoanuts and thick growth of tropical evergreen vegetation, and all are flat, rising only a few feet above the surface of the water. The natives paddle about between these in their dug-out canoes, which are very narrow and light, and are kept right side up by means of a floating outrigger. They cultivate yams and bananas in a lazy kind of way, and each man knows exactly where his patch begins and ends, although there are no marks or fences to define boundaries. Fresh water is scarce on all the islands, so the milk of green cocoanuts is drunk; and when the traveller is thirsty he has only to offer a native boy a morsel of tobacco, and he will walk up a tree and fetch down a load of cocoanuts in less time than it would take to draw a pint of ale from a cask.

These natives take very kindly to the inroads of the white man, and place no obstacle in the way of settlement or trade. I stayed several times with white traders, and in most cases there were but two on each island. The loneliness of the life, they all admitted, was at first very oppressive; but after awhile they grew attached to the lonely surroundings, the quiet, uneventful routine, and all declared that they would never change their circumstances, but would live on to the very end just as they were. This is easily understood, for there are some natures quite unfitted for the bustle and jostle of great cities. The struggle between man and man is painful to them. They see in it only the worst features, and fail to recognise the rest. They long for quietude and regularity; perpetual sunshine, monthly mails, and no telegrams, fogs, or railway trains; and they find these remote spots, Elysiums—realisations of their best ideals.

Fiji is much more settled and civilised than the other coral islands, and it differs from them, too, in formation, having been heaved up by volcanic action, whilst the rest grew in the course of long ages by the labour of the coral insect. The peaks of Levuka, the former capital, tower above the little town, and seem to frown upon the visitor as he glides into the harbour. The residences of the whites are all constructed after the tropical fashion, with broad verandahs and low, flat roofs.

Luva, the capital, is comparatively gay, with its Government House, club, and fine residences. The climate of Fiji is, of course, hot, but the islands are so small as to allow of a cool sea-breeze always permeating through them; and taking into consideration the fruits, the fine weather, the scenery, the lovely and remarkable growth of every kind of tree or shrub, there can be no doubt that Fiji is a fair spot, and well worthy of a visit.

As to the islands of the South Pacific it may be taken for granted that thousands of Englishmen will some day make their homes upon them. Apart from the stormy competition and noisy struggle of the Western and Northern worlds, they will pass their lives in seclusion and quiet regularity, unaffected by the whirlwind of European events, unpained by the sight of hunger and misery and sickness, and almost unaware that the great world consists of anything besides sunshine and happiness.

